# Building Safety Through Orientation: Current Practices of Client and Patient Safety Onboarding in Finland

**DOI:** 10.3390/healthcare14152411

**Published:** 2026-08-05

**Authors:** Saara Ketola, Sini Eloranta, Salla Seppänen

**Affiliations:** 1 Coxa Hospital for Joint Replacement, 33520 Tampere, Finland; 2 Finnish Centre for Client and Patient Safety, Wellbeing Services County of Ostrobothnia, 65130 Vaasa, Finland; sini.eloranta@turkuamk.fi; 3 Department of Health and Wellbeing, Turku University of Applied Sciences, 20520 Turku, Finland; 4 Unit of Social and Health Care, Laurea University of Applied Sciences, 01300 Vantaa, Finland; salla.seppanen@laurea.fi

**Keywords:** patient safety, staff orientation, safety culture, healthcare professionals, competency, implementation

## Abstract

Comprehensive orientation is essential for ensuring client and patient safety, strengthening safety culture, and supporting workforce competence in social and health care. Although orientation is recognized as an important patient safety intervention, little is known about how comprehensively client and patient safety topics are incorporated into orientation programs. This study evaluated the extent to which client and patient safety topics were incorporated into orientation programs across Finnish wellbeing services counties. Data: A cross-sectional electronic survey was conducted in 2024. The questionnaire was based on the Finnish national client and patient safety orientation framework and included thirty-nine orientation topics covering safety management, occupational safety, safety culture, risk management, medication safety, infection prevention, information security, and emergency preparedness. Responses were requested from ten predefined social and health care service areas in each of Finland’s 23 wellbeing services counties. Fourteen counties participated, yielding 107 responses. Results: Traditional client and patient safety topics were widely incorporated into orientation programs. The highest affirmative responses concerned individual job responsibilities, confidentiality, processing of client and patient information, occupational safety, medication competence, and incident reporting (all 96–99%). In contrast, psychological safety (58%), second victim practices (14%), self-monitoring programs and plans (42% and 54%), safety culture assessment (41%), and client and patient safety in digital and remote services (62%) were incorporated less frequently. Conclusions: This study provides a national overview of client and patient safety orientation practices based on responses from participating Finnish wellbeing services counties. Traditional client and patient safety topics were widely incorporated into orientation programs, whereas psychological safety, second victim support, self-monitoring, and digital safety were less consistently represented. The findings suggest that implementation of patient safety priorities is uneven across safety domains, with newer organizational and systems-oriented topics appearing less embedded in orientation than traditional clinical safety topics. Orientation may therefore be viewed not merely as an onboarding activity but also as a strategic mechanism for implementing patient safety priorities within healthcare organizations.

## 1. Introduction

Patient safety education and workforce competence development are increasingly recognized as core components of international patient safety policy. The World Health Organization (WHO) has promoted patient safety competencies through the Patient Safety Curriculum Guide and, more recently, through the Global Patient Safety Action Plan 2021–2030, which emphasizes education, workforce capability, safety culture, leadership, and learning systems as prerequisites for safer care [1]. Similar competency-based frameworks have been developed in several countries, including Australia [2], Canada [3], the United Kingdom [4], and the Nordic countries [5], to support the integration of patient safety principles into professional education and continuing workforce development.

In Finland, patient safety is addressed through a broader concept of client and patient safety, reflecting the integrated governance of healthcare and social welfare services. The Finnish National Client and Patient Safety Strategy 2022–2026 aligns closely with the WHO Global Patient Safety Action Plan while extending its scope beyond healthcare to include social services [6]. The strategy emphasizes safety culture, proactive risk management, evidence-based management, workforce competence, patient and client involvement, and continuous organizational learning. Particular emphasis is placed on systematic orientation and onboarding processes as essential mechanisms for ensuring safe practice and supporting workforce retention in changing service environments.

Client and patient safety forms a fundamental basis of health and social care. It refers to the principles and practices used to ensure the safety of care and services. This includes a safety culture as well as processes and procedures designed to reduce risks and prevent preventable harm [1,6]. Safety is grounded in professional competence, ethics, good governance and leadership, and evidence-based knowledge. Client and patient safety is a prerequisite for high-quality, effective, and equitable services and should not be viewed as a separate component of the service system [7].

Adverse events and patient safety incidents continue to present significant challenges. In Finland, the annual costs associated with patient safety incidents are estimated at one billion euros. Up to 15% of healthcare expenditure is related to correcting errors and resulting harm [8]. In 2025, 10,800 patient injury claims were submitted to the Finnish Patient Insurance Centre, and compensation payments exceeded thirty million euros [9]. Globally, patient safety incidents have been estimated to reduce economic growth by 0.7% annually [10]. These figures underline the need for initiative-taking strategies and systematic prevention of errors and harm.

The social and health care sector is the largest employer in Finland. At the end of 2023, approximately 372,600 people were employed in the sector [11]. Of all licensed health and social care professionals, 3.7% were born outside Finland and 2.3% were foreign citizens [12]. The Finnish Government’s Good Work Programme 2024–2027 aims to ensure workforce sufficiency and strengthen the attractiveness and retention of the sector through measures such as increasing education opportunities, improving task allocation, supporting occupational well-being, reducing unnecessary work tasks, and expanding international recruitment [13].

Adequate professional competence, continuous education, and a safe working environment are essential prerequisites for patient safety [1,14,15]. Employers are obligated to ensure staff competence and provide adequate orientation so that employees understand workplace practices, job responsibilities, and associated safety risks [16,17]. Orientation is a principal component of proactive safety management and lifelong professional learning [6].

Orientation is typically associated with the beginning of employment. Research suggests that orientation programs improve newly graduated professionals’ competence, clinical skills, and decision-making abilities [18]. However, experienced employees also require orientation when changing workplaces or units. Additional orientation is necessary when introducing new equipment or software or when employees return after a prolonged absence [16,17,19]. To ensure safety, orientation should include information about the work environment, safety procedures, and reporting systems for adverse events and near misses. Multiprofessional orientation has been shown to improve both commitment to the workplace and patient safety [20].

An effective orientation program is systematic, individualized, and evidence-based, combining education, clinical support, and continuous monitoring [21]. Orientation should consider the duration of employment, professional competence, experience, educational background, and language and cultural background. Supervisors are responsible for the content of orientation programs, although practical implementation may be delegated. Competence development is gradual and may take years [16,17,19]. Orientation programs lasting several months or even a year have been developed for newly graduated physicians and nurses [22,23]. As international recruitment increases, the importance of orientation is further emphasized. Successful integration of internationally recruited professionals requires carefully planned orientation processes [24,25].

## 2. Methods

The purpose of this study was to assess the current state of client and patient safety orientation practices in Finnish wellbeing services counties. The study aimed to generate baseline information to support organizational development during a period of major restructuring and changing job responsibilities and prior to the implementation of the national Client and Patient Safety Orientation Model.

Despite the widely acknowledged importance of orientation for patient safety, workforce competence, and organizational effectiveness, research evidence regarding the implementation of client and patient safety orientation programs remains scarce. Previous studies have mainly focused on orientation within individual organizations or professional groups, whereas no national assessment of client and patient safety orientation practices has been conducted in Finland.

In Finland, the concept of client and patient safety is used instead of patient safety alone, reflecting the integration of healthcare and social services as well as the broader perspective on safety across service systems. Finland has a publicly funded social and health care system. Since the Finnish health and social services reform of 2023, responsibility for organizing public healthcare, social services, and rescue services has been transferred to regional authorities referred to as wellbeing services counties. These counties are responsible for organizing both social and health care services for their populations, including primary care, specialized medical care, mental health services, long-term care, disability services, child welfare services, and emergency care. The integration of social and health care within a single organizational structure creates a unique context for examining orientation practices related to client and patient safety.

The study was a descriptive cross-sectional survey. Data were collected in 2024 using an electronic questionnaire distributed to all Finnish wellbeing services counties. Finland’s health and social services system is organized through twenty-one wellbeing services counties, while the City of Helsinki and the autonomous Åland Islands independently hold corresponding organizational responsibility, resulting in a total of twenty-three public service organizers. The questionnaire was sent to individuals responsible for client and patient safety within the counties, who were asked to forward it to representatives from ten specified specialties (Table 1). The selected service sectors were chosen to represent the diversity of Finnish health and social care services, including both primary and specialized care, adult and pediatric services, somatic and psychiatric care, preventive services, emergency care, long-term care, and community-based services. Each wellbeing services county was therefore expected to submit ten completed responses. The invitation requested that the questionnaire be completed by the person responsible for orientation within the selected specialty or service area (e.g., the frontline manager responsible for staff orientation). The final selection of respondents was made locally by each wellbeing services county.

All 23 Finnish wellbeing services counties were invited to participate. The study was therefore designed as a national census rather than a sample survey. Participation was voluntary, and no replacement sampling was performed for non-responding counties.

The questionnaire was based on the national orientation model for client and patient safety developed by the Finnish Centre for Client and Patient Safety [26] and included thirty-nine themes (Table 2). Data were collected before the publication and implementation of the orientation model to provide baseline information on orientation practices prior to implementation. Although the questionnaire was based on a nationally developed orientation framework and reviewed by subject matter experts, it was not formally psychometrically validated. Therefore, the findings should be interpreted as a descriptive assessment of the implementation of nationally recommended orientation topics rather than as measurements of underlying constructs. The questionnaire was designed to assess whether nationally recommended orientation topics were included in local orientation programs. Therefore, the items represented predefined content areas derived from the national orientation model rather than latent constructs requiring psychometric scale development. The national orientation model served as the conceptual framework for the survey rather than the object of evaluation. The unit of analysis was the individual questionnaire response, each representing one predefined specialty or service area within a participating wellbeing services county.

The purpose of the study was not to compare wellbeing services counties. Because the study was descriptive, county-specific results were not reported.

In Finland, self-monitoring constitutes the primary mechanism for quality assurance and risk management in health and social care services. Self-monitoring refers to the systematic activities through which service organizers and providers oversee, evaluate, and continuously improve their operations to ensure legality, safety, quality, continuity, accessibility, and equality of services. It is an initiative-taking approach that includes identifying risks, addressing deficiencies, and implementing corrective actions before adverse events occur. In addition to organizational responsibilities, all social and health care professionals are legally obliged to report identified risks and unsafe practices to their employer.

The selected specialties and service areas were chosen to represent the breadth of Finnish health and social care services across the continuum of care. Five respondents were sought from hospital-based services and five from community-based services in each wellbeing services county. Hospital-based services included Internal Medicine, Surgery, Pediatrics, Psychiatry, and Emergency Care, representing the largest medical specialties as well as both somatic and mental health services for adult and pediatric populations. Community-based services included Primary Health Care Centers, Maternity, Child Health and School Health Clinics, Older Adult Care Services, Child Protection Services, and Disability and Intellectual Disability Services. Together, these services encompass preventive care, primary care, long-term care, home-based services, social services, and specialized health care. The selection was intended to capture variation in patient populations, care settings, and professional practices rather than to compare specialties with one another.

### 2.1. Statistical Analyses

Statistical analyses were descriptive in nature. Results are presented as percentages of affirmative responses and corresponding 95% confidence intervals (CIs) among all respondents. Confidence intervals for proportions were calculated using the Wilson score method, which is recommended over the standard Wald interval because of its superior coverage properties for binomial data, particularly when sample sizes are relatively small or observed proportions are close to the boundaries of 0 or 1.

The study was designed as a national census rather than a sampled survey, thus no statistical weighting or imputation for non-response was performed. The results therefore describe the participating wellbeing services counties and should be interpreted as indicative of current national orientation practices rather than fully representative of all Finnish wellbeing services counties.

### 2.2. Survey Development and Content Validity

The survey instrument was developed specifically for this study based on the national Client and Patient Safety Orientation Model published by the Finnish Centre for Client and Patient Safety. The orientation model was developed as part of the implementation of the Finnish Client and Patient Safety Strategy 2022–2026 [6] coordinated by the Ministry of Social Affairs and Health. Survey items corresponded directly to the orientation topics included in the national model. Before dissemination, the questionnaire was reviewed by experts involved in the development of the orientation model. Because the survey was designed as a descriptive implementation assessment rather than a psychometric instrument measuring latent constructs, formal validation procedures, such as construct validity testing or reliability analyses, were not performed. It identifies key themes considered essential for ensuring client and patient safety competence among newly recruited personnel in Finnish social and health care organizations. The resulting questionnaire consisted of thirty-nine orientation topics grouped into twelve thematic domains, including orientation planning, occupational safety and wellbeing, safety culture, risk management, patient autonomy, incident reporting, infection prevention, medication safety, medical device safety, information security, emergency preparedness, and physical security. Content validity was established through expert-based development. The orientation model had been developed through a national expert collaboration, i.e., by experts in client and patient safety, healthcare management, social welfare services, occupational safety, medication safety, and quality management. Prior to data collection, the questionnaire was reviewed by experts from the Finnish Centre for Client and Patient Safety and representatives involved in national patient safety strategy implementation to ensure relevance, clarity, and consistency with current Finnish regulations and policy requirements.

### 2.3. Ethics Statement

The study did not involve patients, interventions, biological samples, or personal health data and therefore did not fall within the scope of the Finnish Medical Research Act (488/1999, Chapter 1, Section 2). This study did not involve intervention in the physical integrity of participants, exceptional psychological burden, participation of minors without parental consent, exposure to exceptionally strong stimuli, or risks exceeding those encountered in everyday life. Consequently, approval by a medical research ethics committee was not required under Finnish legislation. It was conducted in accordance with the Finnish National Board on Research Integrity (TENK) guidelines on research involving human participants. Participants were informed about the study aims, implementation, handling of responses, and publication of results when the electronic questionnaire was distributed. Participation was voluntary, and completion of the questionnaire was regarded as informed consent [27,28].

The study was conducted as part of the monitoring of the implementation program of the Finnish National Client and Patient Safety Strategy coordinated by the Ministry of Social Affairs and Health [6].

### 2.4. Data Protection Statement

No personal health data or other sensitive personal data were collected. Data were analyzed and reported at an aggregated level, and individual respondents, organizations, and wellbeing services counties cannot be identified from the published results. Data processing complied with applicable Finnish and European Union data protection legislation.

## 3. Results

Responses were received from fourteen wellbeing services counties (14/23) within ten specialties (Older Adult Care Services, Surgical Specialty, Child Protection Services, Pediatric Specialty, Maternity, Child Health and School Health Clinics, Psychiatric Specialty, Emergency Department, Internal Medicine Specialty, Primary Health Care Center, Disability and Intellectual Disability Services). A total of 107 response sets were obtained, distributed relatively evenly across specialties. Percentages are presented together with their corresponding 95% Wilson confidence intervals (Table 2). Because the number of responses within individual service sectors was limited, analyses were performed using the combined national dataset. The aim of the study was to provide an overall assessment of the incorporation of client and patient safety topics into orientation programs rather than to compare individual specialties or care settings.

The highest proportions of affirmative responses concerned orientation related to employees’ job descriptions and responsibilities, handling of client and patient information and data protection, and confidentiality and professional secrecy. Basic information on client and patient safety was also widely included in orientation programs. Ninety percent or more affirmative responses were obtained in sixteen of the thirty-nine themes.

In contrast, five themes received affirmative responses from less than 50% of respondents: self-monitoring programs, safety culture, second victim practices, and radiation and chemical safety.

Second victim practices were the least frequently included topic. They were addressed in orientation programs in only 14%. Assessment and measurement of safety culture were included in orientation in 41%, whereas practices aimed at strengthening safety culture were included in 75%. Psychological safety was included in 58% of orientation programs and occupational well-being measurement in 52%.

Self-monitoring programs and self-monitoring plans were included in orientation in 42% and 54% of the specialties or service areas, respectively. Considerable variation existed between specialties (Supplementary Table S1).

Patients’ right to self-determination and preventive working methods and restrictive measures were included in orientation in 79% and 82% of the selected specialties or service areas, respectively.

Hand hygiene was comprehensively included in orientation in 89%, although variation still existed. Client and patient feedback systems were included in orientation in 83%. The safety of digital and remote services was included in orientation programs in 62% of the specialties overall.

## 4. Discussion

This study examined the extent to which client and patient safety topics are incorporated into orientation programs across Finnish wellbeing services counties. We identified both well-established and less frequently included client and patient safety orientation topics. The highest affirmative response rates were observed for topics directly linked to everyday clinical practice and regulatory compliance, including individual job responsibilities, processing of client and patient data and data protection, confidentiality and professional secrecy, occupational safety, basic principles of client and patient safety, medication competence verification, medication permits, incident reporting, and access control systems. The findings suggest that implementation of national patient safety priorities is uneven. Topics that have long been embedded in professional education, regulation, and organizational practice are widely represented in orientation programs, whereas newer safety concepts appear to be less consistently integrated. This pattern indicates that current orientation practices primarily support organizational operational readiness by ensuring that employees possess the knowledge required to fulfil statutory responsibilities and perform essential safety-related tasks. Client and patient safety should not, however, be viewed solely as compliance with regulations; rather, it should be understood as a broader concept that encompasses elements such as safety culture, as highlighted in the WHO Global Patient Safety Action Plan 2021–2030 [1]. In addition, contemporary patient safety frameworks increasingly emphasize culture, learning systems, and resilience as essential components of safe healthcare rather than focusing solely on procedural compliance. For example, psychological safety received affirmative responses from 58% of respondents. One possible explanation is that psychological safety is often perceived as an aspect of leadership and team culture rather than as an explicit orientation topic. Nevertheless, growing evidence links psychological safety to speaking up, learning from errors, teamwork, and patient outcomes. Introducing psychological safety already during orientation has been suggested to strengthen new employees’ willingness to report hazards and participate actively in safety improvement [29].

The low inclusion of radiation and chemical safety may reflect the fact that these topics are not relevant to all specialties and service areas. Therefore, these findings should be interpreted with caution and should not necessarily be regarded as deficiencies in orientation programs. The comprehensive inclusion of medication competence in orientation programs represents a positive finding. Given the substantial burden of medication-related harm worldwide, ensuring that healthcare professionals are familiar with medication plans, competence requirements, and medication permits at the start of employment may contribute to safer medication practices and risk reduction.

Particularly noteworthy was the limited inclusion of second victim practices in orientation programs. This finding may reflect the recent recognition of second victim phenomena within healthcare organizations and the absence of standardized organizational support pathways in many institutions. Although adverse event reporting systems are generally well established, structured support for professionals involved in incidents appears considerably less mature. The previous literature suggests that the absence of such support may be associated with reduced staff well-being, lower reporting activity, burnout, and workforce attrition. Adverse events may have significant psychological consequences not only for patients but also for healthcare professionals involved in the event. They may experience shame, guilt, fear, regret, and psychological distress, potentially leading to sick leave or even leaving the profession [30,31]. Rapid and non-punitive support might prevent prolonged symptoms and deterioration of professional self-esteem. Support from colleagues is essential. Effective second victim practices also facilitate open and honest discussions about adverse events with colleagues, patients, and families [32,33].

Safety should not be assessed solely through past adverse events; current operational processes and future risks must also be evaluated. Within this framework, self-monitoring is a key risk management tool [34,35]. The relatively low inclusion of self-monitoring programs and plans in orientation was noteworthy. Inclusion in orientation remained notably below the national strategic target level of 80% [6]. Because the survey was conducted during the early implementation phase of the Finnish healthcare reform, many organizations may still have been developing their self-monitoring structures and had not yet fully integrated them into orientation programs. Although self-monitoring is a Finnish regulatory concept, the underlying principles are aligned with internationally recognized approaches to clinical governance and proactive risk management. The findings suggest that organizational and system-oriented safety practices may not yet be as firmly embedded in orientation programs as more traditional clinical safety topics. This observation may indicate that the implementation of newer safety management concepts remains at an earlier stage than the implementation of long-established patient safety practices.

Digital and remote service safety also received fewer affirmative responses than expected. This may indicate that digital competencies are still viewed primarily as technical system training rather than as patient safety competencies. However, the increasing use of remote consultations, digital monitoring, and artificial intelligence requires orientation programs to address not only system usability but also information security, communication challenges, clinical risks, and patient safety in digital environments.

These findings may have broader international relevance, as healthcare systems worldwide are undergoing rapid digital transformation. The World Health Organization has highlighted digital health as a key component of strengthening health systems and improving access to care [36]. Digital technologies are promoted as solutions to workforce shortages, growing service demands, and healthcare system sustainability. At the same time, the safe implementation of digital technologies depends on the competencies of frontline professionals. The relatively limited attention paid to digital safety in orientation programs may reflect a potential gap between digital health policy ambitions and the orientation content currently provided to healthcare professionals. Strengthening digital safety competencies during orientation could support the implementation of both national and international patient safety and digital health strategies.

Overall, the findings suggest that Finnish wellbeing services counties have largely succeeded in establishing orientation practices that support compliance with statutory obligations and safe day-to-day operations. However, strategic themes related to safety culture, workforce well-being, digital transformation, and proactive safety management were less consistently integrated into orientation programs. Beyond describing the prevalence of orientation topics, these findings provide new insights into how different dimensions of client and patient safety are translated into orientation practice. The results indicate that implementation of patient safety priorities is not uniform across safety domains and that newer organizational and system-oriented concepts may require more active integration into orientation programs. Consequently, orientation programs appear to reflect both the strengths and the remaining implementation challenges of contemporary patient safety systems. These findings contribute to the international literature by demonstrating that implementation of patient safety priorities may vary considerably across safety domains, highlighting the gap between national patient safety frameworks and their practical integration into workplace orientation.

### Scientific Contribution of the Study

Whereas previous studies have predominantly examined orientation within single organizations or professional groups, this study examines the incorporation of client and patient safety topics into orientation programs across participating Finnish wellbeing services counties. The survey evaluates orientation against a nationally developed framework based on the Finnish Client and Patient Safety Strategy, thereby linking strategic policy objectives with their implementation in workplace orientation.

Importantly, the findings demonstrate that implementation of patient safety priorities is not uniform across safety domains. While traditional clinical and regulatory safety topics were widely incorporated into orientation programs, organizational and systems-oriented topics—including psychological safety, second victim support, self-monitoring, and digital safety—were considerably less represented. These findings provide baseline information that may support future evaluation of national patient safety strategy implementation and identify areas where further integration into orientation programs may be needed.

Although the survey was conducted within the Finnish healthcare system, the findings are also relevant internationally because many healthcare systems are currently implementing national patient safety strategies aligned with the WHO Global Patient Safety Action Plan. Understanding which patient safety topics become integrated into routine orientation—and which remain less consistently incorporated—may help other countries identify similar implementation challenges.

## 5. Conclusions

This study provides a national baseline based on responses from participating Finnish wellbeing services counties, describing the extent to which key client and patient safety topics have been incorporated into orientation across different health and social care settings. Such baseline information is essential for monitoring implementation of national patient safety strategies and evaluating future policy interventions. Traditional clinical safety topics appear to be well established, while newer organizational and system-based dimensions of patient safety have not yet been consistently embedded into everyday orientation practices. These findings highlight an important gap between national patient safety frameworks and their practical implementation in workplace orientation and identify priorities for healthcare organizations, managers, educators, and policymakers.

Future national surveys would provide valuable information on the implementation of the national orientation model and the evolution of orientation practices over time. Further research should extend beyond assessing the presence of orientation topics to evaluate the quality, intensity, educational methods, competence assessment, and implementation fidelity of orientation programs. Studies with larger samples could also explore variation across service sectors, specialties, and organizational contexts. Subsequent longitudinal studies could examine whether comprehensive orientation is associated with improvements in safety culture, workforce retention, employee well-being, and client and patient safety outcomes.

## 6. Strengths and Limitations

A strength of this study is that all 23 Finnish wellbeing services counties were invited to participate, making the survey national in scope rather than based on a selected sample. The participating counties represented different geographical regions and both university and non-university hospital districts, providing a broad overview of orientation practices across the Finnish healthcare system. The study addressed both healthcare and social welfare services, reflecting the integrated service structure unique to the Finnish client and patient safety framework.

The study also has limitations. Responses were obtained from only 14 of the 23 wellbeing services counties. Consequently, the findings may be affected by non-response bias. It is possible that counties with more established orientation practices or greater interest in client and patient safety were more likely to participate, while organizations facing greater implementation challenges may be underrepresented. Conversely, counties with well-developed orientation programs may also have considered participation less urgent. Because the characteristics of non-responding counties are unknown, the direction and magnitude of any potential bias cannot be determined. Therefore, the findings should be interpreted as reflecting the orientation practices of participating wellbeing services counties rather than providing precise national prevalence estimates. However, the study offers the first nationwide overview of client and patient safety orientation practices in Finland and identifies important areas requiring further attention regardless of the exact prevalence. Yet, responses were received from all ten predefined service sectors included in the study. This broad coverage across different social and healthcare settings strengthens the transferability of the findings across different organizational contexts. This enabled the study to capture orientation practices across both healthcare and social welfare settings.

The survey was based on the national Client and Patient Safety Orientation Model developed as part of the implementation of the Finnish Client and Patient Safety Strategy 2022–2026. Consequently, the questionnaire content reflected nationally recommended orientation topics rather than locally developed practices. The survey instrument was specifically developed for this study and was not subjected to formal validation. However, the questionnaire was derived from a nationally developed orientation framework and reviewed by experts involved in client and patient safety development.

Several survey items reflect characteristics of the Finnish social and health care system and may not be directly transferable to other countries. These include the concepts of the self-monitoring program and plan, which are statutory quality and safety management tools required under Finnish social and health care legislation.

The survey assessed whether specific topics were included in orientation programs but did not evaluate the quality, duration, educational methods, or effectiveness of orientation practices. Consequently, the findings describe the coverage of orientation content rather than the actual competence achieved by employees following orientation. Furthermore, the survey relied on self-reported information provided by respondents and may therefore be subject to reporting bias.

The study was conducted during the early years of the Finnish wellbeing services counties reform, which transferred responsibility for health, social welfare, and rescue services from municipalities to newly established regional authorities in 2023. The participation rate may partly reflect the extensive organizational reforms and workload pressures associated with the establishment of the wellbeing services counties during the study period. Also, orientation practices were likely still evolving.

## Figures and Tables

**Table 1:** Social and healthcare specialties/service areas invited to participate in the client and patient safety orientation survey from each wellbeing services county.

Child Protection Services
Disability and Intellectual Disability Services
Emergency Department
Internal Medicine Specialty
Maternity, Child Health, and School Health Clinics
Older Adult Care Services
Pediatric Specialty
Primary Health Care Center
Psychiatric Specialty
Surgical Specialty

**Table 2:** Inclusion of client and patient safety topics in orientation programs: proportion of affirmative responses (%) and 95% confidence intervals (Wilson method) by topic area (N = 107).

Topics Related to Orientation Planning, Implementation, Monitoring and Evaluation	Yes (%)	95% Wilson CI
Organization’s Mission and Vision	66%	57.0–74.6%
Organizational Values	83%	75.0–89.1%
Basic Principles of Client and Patient Safety	97%	92.1–99.0%
Employer and Employee Responsibilities and Obligations	93%	87.1–97.0%
Orientation Plan, Follow-up, and Evaluation	92%	84.8–95.5%
**Occupational Safety and Employee Well-Being**		
Individual Job Description and Areas of Responsibility	99%	94.9–99.8%
Psychological Safety	58%	48.5–66.9%
Second Victim Practices	14%	8.7–21.9%
Measurement of Occupational Well-being	52%	43.0–61.6%
Occupational Safety	98%	93.4–99.5%
Ergonomics	75%	65.8–82.0%
**Safety Culture and Workplace Safety**		
Assessment and Measurement of Safety Culture	41%	32.2–50.6%
Practices to Strengthen Safety Culture	74%	64.8–81.2%
Self-Monitoring Program	42%	33.1–51.5%
Self-Monitoring Plan	54%	44.8–63.3%
Monitoring and Measurement of Client and Patient Safety	79%	69.8–85.2%
**Risk Management**		
Risk Management	89%	84.1–93.5%
**Patient Autonomy and Restrictive Practices**		
Client and Patient Self-Determination Rights	79%	69.8–85.2%
Preventive Working Approaches and Restrictive Measures	81%	72.9–87.6%
Client and Patient Feedback Systems	83%	75.0–89.1%
**Incident Reporting and Statutory Notification Procedures**		
Statutory Reporting Obligations and Rights	71%	61.8–78.8%
Adverse Event, Safety Incident and Near-Miss Reporting	96%	90.8–98.5%
Reporting and Management of Serious Adverse Events	92%	84.5–95.5%
Documentation and Processing of Reports	90%	82.5–94.2%
**Infection Prevention and Control**		
Hand Hygiene	89%	81.4–93.5%
Guidelines for Employee Appearance	92%	84.8–95.5%
Required Employee Vaccinations	94%	88.3–97.4%
**Medication Safety and Competency**		
Medication Management Plan	89%	81.4–93.5%
Verification of Medication Competence	96%	90.8–98.5%
Medication Permits and Competency Demonstrations	96%	90.8–98.5%
**Medical Device Safety and Competency**		
Verification of Medical Device Competence	88%	80.3–92.8%
Medical Device Permits and Competency Demonstrations	70%	60.9–78.0%
**Chemical and Radiation Safety**		
Radiation Safety	23%	16.4–32.2%
Chemical Safety	48%	38.5–57.0%
**Information Security and Data Protection**		
Processing of Client and Patient Data and Data Protection	99%	94.9–99.8%
Client and Patient Safety in Digital and Remote Services	62%	52.2–70.3%
Confidentiality and Professional Secrecy	99%	94.9–99.8%
**Emergency Preparedness and Response**		
Safety in Emergency and Exceptional Situations (e.g., resuscitation, poisoning, threatening situations)	92%	84.8–95.5%
**Physical Security and Access Control**		
Access Control and Alarm Systems	96%	90.8–98.5%

## Data Availability

The anonymized dataset supporting the findings of this study is not publicly available because it was collected for the monitoring of the implementation program of the Finnish National Client and Patient Safety Strategy and contains organizational-level information. The data have been provided to the journal during the review process for verification purposes.

## Supplementary Materials

The following supporting information can be downloaded at https://www.mdpi.com/article/10.3390/healthcare14152411/s1. Table S1. Results by specialties/service areas.
