# Supplementary material for: Building Safety Through Orientation: Current Practices of Client and Patient Safety Onboarding in Finland
Source: Healthcare (Basel). 2026 Aug 5;14(15):2411. doi: 10.3390/healthcare14152411 (PMC13465449; doi:10.3390/healthcare14152411)
Supplement: Supplementary file 1 [file healthcare-14-02411-s001.zip › healthcare-4380714-supplementary.pdf]

Supplementary Table S1: Results by specialties/service areas

	Older Adult Care Services	Surgical Specialty	Child Protection Services	Pediatric Specialty	Maternity, Child Health and School Health Clinics	Psychiatric Specialty	Emergency Department	Internal Medicine Specialty	Primary Health Care Center	Disability and Intellectual Disability Services	
	**Yes**	**Yes**	**Yes**	**Yes**	**Yes**	**Yes**	**Yes**	**Yes**	**Yes**	**Yes**	**RANGE (%)**
Organization's Mission and Vision	77 %	50 %	20 %	57 %	57 %	78 %	71 %	80 %	76 %	57 %	20-80
Organizational Values	96 %	63 %	40 %	71 %	86 %	89 %	71 %	100 %	88 %	86 %	40-96
Basic Principles of Client and Patient Safety	96 %	88 %	100 %	100 %	100 %	100 %	86 %	100 %	100 %	100 %	86-100
Employer and Employee Responsibilities and Obligations	96 %	75 %	100 %	100 %	93 %	100 %	71 %	100 %	100 %	86 %	71-100
Orientation Plan, Follow-up and Evaluation	96 %	88 %	100 %	100 %	79 %	89 %	100 %	100 %	88 %	86 %	79-100
Individual Job Description and Areas of Responsibility	96 %	100 %	100 %	100 %	100 %	100 %	100 %	100 %	100 %	100 %	96-100
Psychological Safety	54 %	57 %	60 %	43 %	64 %	89 %	43 %	40 %	59 %	71 %	40-89
Second Victim Practices	8 %	38 %	0 %	14 %	21 %	22 %	29 %	0 %	6 %	14 %	0-38
Measurement of Occupational Well-being	46 %	25 %	20 %	43 %	50 %	78 %	29 %	60 %	71 %	71 %	20-78
Occupational Safety	100 %	75 %	100 %	100 %	100 %	100 %	100 %	100 %	100 %	100 %	75-100
Ergonomics	92 %	38 %	60 %	86 %	79 %	67 %	43 %	80 %	65 %	100 %	38-100

Assessment and Measurement of Safety Culture	35 %	13 %	60 %	43 %	36 %	100 %	43 %	20 %	41 %	29 %	13-100
Practices to Strengthen Safety Culture	77 %	43 %	100 %	100 %	57 %	100 %	71 %	40 %	71 %	86 %	40-100
Self-Monitoring Program	62 %	38 %	60 %	14 %	43 %	11 %	0 %	0 %	47 %	86 %	0-86
Self-Monitoring Plan	96 %	25 %	80 %	29 %	36 %	22 %	0 %	20 %	65 %	86 %	0-96
Monitoring and Measurement of Client and Patient Safety	88 %	50 %	100 %	71 %	71 %	100 %	29 %	80 %	88 %	86 %	29-100
Risk Management	96 %	75 %	100 %	57 %	86 %	100 %	100 %	60 %	88 %	100 %	57-100
Client and Patient Self-Determination Rights	96 %	38 %	100 %	71 %	64 %	100 %	57 %	80 %	71 %	100 %	38-100
Preventive Working Approaches and Restrictive Measures	96 %	29 %	100 %	71 %	79 %	100 %	86 %	60 %	71 %	100 %	29-100
Client and Patient Feedback Systems	88 %	50 %	80 %	71 %	79 %	100 %	71 %	100 %	88 %	86 %	50-100
Statutory Reporting Obligations and Rights	88 %	50 %	80 %	43 %	71 %	89 %	57 %	20 %	76 %	57 %	20-89
Adverse Event, Safety Incident and Near-Miss Reporting	100 %	63 %	100 %	86 %	100 %	100 %	100 %	100 %	100 %	100 %	63-100
Reporting and Management of Serious Adverse Events	92 %	63 %	100 %	86 %	86 %	100 %	100 %	80 %	100 %	100 %	63-100
Documentation and Processing of Reports	96 %	63 %	80 %	86 %	86 %	100 %	71 %	100 %	94 %	100 %	63-100
Hand Hygiene	96 %	88 %	60 %	100 %	57 %	89 %	86 %	100 %	100 %	100 %	60-100
Guidelines for Employee Appearance	100 %	75 %	80 %	100 %	79 %	100 %	71 %	100 %	94 %	100 %	71-100
Required Employee Vaccinations	92 %	88 %	80 %	100 %	93 %	100 %	100 %	100 %	100 %	86 %	80-100
Medication Management Plan	96 %	50 %	100 %	86 %	79 %	100 %	100 %	100 %	88 %	100 %	50-100
Verification of Medication Competence	100 %	75 %	100 %	100 %	93 %	100 %	100 %	100 %	94 %	100 %	75-100
Medication Permits and Competency Demonstrations	100 %	75 %	100 %	100 %	93 %	100 %	100 %	100 %	94 %	100 %	75-100
Verification of Medical Device Competence	92 %	75 %	80 %	100 %	71 %	78 %	100 %	100 %	94 %	100 %	71-100
Medical Device Permits and Competency Demonstrations	81 %	57 %	80 %	86 %	43 %	67 %	100 %	100 %	59 %	71 %	43-100
Radiation Safety	15 %	63 %	0 %	43 %	7 %	11 %	14 %	40 %	41 %	14 %	0-63
Chemical Safety	36 %	50 %	40 %	71 %	43 %	44 %	29 %	40 %	82 %	43 %	29-82
Processing of Client and Patient Data and Data Protection	100 %	88 %	100 %	100 %	100 %	100 %	100 %	100 %	100 %	100 %	88-100
Client and Patient Safety in Digital and Remote Services	46 %	29 %	80 %	57 %	71 %	89 %	29 %	80 %	82 %	57 %	29-89
Confidentiality and Professional Secrecy	100 %	88 %	100 %	100 %	100 %	100 %	100 %	100 %	100 %	100 %	88-100

Safety in Emergency and Exceptional Situations (e.g., resuscitation, poisoning, threatening situations)	96 %	75 %	100 %	86 %	79 %	100 %	86 %	100 %	94 %	100 %	75-100
Access Control and Alarm Systems	92 %	100 %	100 %	100 %	86 %	100 %	100 %	100 %	100 %	100 %	86-100
